# Predictive value of the product term BRI × carotid plaque thickness for stroke and transient ischemic attack: a prospective cohort study

**DOI:** 10.3389/fneur.2025.1622941

**Published:** 2025-09-17

**Authors:** Leilei Yan, Enpeng Xing, Chunhua He, Zedong Zhang

**Affiliations:** ^1^Department of Vascular Surgery, Binzhou People's Hospital, Affiliated to Shandong First Medical University, Binzhou, China; ^2^Department of Ultrasound Medicine, Binzhou People's Hospital, Affiliated to Shandong First Medical University, Binzhou, China

**Keywords:** stroke, transient ischemic attack, carotid plaque, body roundness index, interaction, predictive model

## Abstract

**Background:**

Carotid plaque thickness and BRI are each associated with an increased risk of stroke. However, the value of their interaction in predicting stroke remains unclear. This study aimed to investigate the predictive performance of maximum carotid plaque thickness, BRI, and their interaction for the occurrence of stroke or TIA.

**Methods:**

In this prospective cohort study, 230 elderly Chinese adults were enrolled. Baseline measurements included maximum carotid plaque thickness and BRI, and an interaction term was calculated. Participants were followed for 1 year, during which the incidence of stroke or TIA was recorded. Multivariable logistic regression was used to assess the predictive value of each variable. Receiver operating characteristic curve analysis with 95% confidence intervals was conducted to determine the area under the curve (AUC) for model performance, and internal validation using bootstrap resampling (*B* = 1,000) was performed to correct for potential optimism.

**Results:**

Both maximum plaque thickness (3.305 ± 0.515 mm vs. 2.245 ± 0.820 mm, *p* < 0.001) and BRI (4.872 ± 1.240 vs. 3.751 ± 0.916, *p* < 0.001) were significantly higher in the stroke group than in the non-stroke group. Logistic regression analysis showed that maximum plaque thickness (Full multivariable adjustment: OR = 3.619, 95%CI: 1.781–7.355, *p* = 0.00038) and BRI (Full multivariable adjustment: OR = 3.116, 95% CI: 1.784–5.444, *p* = 0.00006) were both independent predictors. ROC analysis revealed that the interaction term yielded the highest AUC (0.9192, 95% CI: 0.8772–0.9612), compared with maximum plaque thickness (0.8819, 95% CI: 0.8353–0.9285) and BRI (0.7632, 95% CI: 0.6266–0.8997). Statistical comparisons indicated that the interaction model significantly outperformed BRI, while its advantage over maximum plaque thickness was numerically higher but did not reach statistical significance, likely due to the limited number of events. After bootstrap correction (*B* = 1,000), the optimism-corrected AUC of the interaction model was 0.897 (95% CI: 0.788–0.954).

**Conclusion:**

Both maximum carotid plaque thickness and BRI independently predict the risk of stroke and TIA after adjusting for confounders. Their interaction further improves predictive performance. Combined assessment of these indicators may optimize early stroke risk stratification and warrants further validation in clinical practice.

## 1 Introduction

Stroke remains a leading cause of death and long-term disability across the globe, while transient ischemic attack (TIA) is widely considered an important warning event preceding stroke (1, 2). In recent years, carotid atherosclerosis has been increasingly recognized as a major contributor to stroke pathogenesis, with both the morphology and structural features of carotid plaques emerging as key indicators of cerebrovascular risk (3–5). At the same time, researchers have paid growing attention to how body fat distribution may influence vascular health. The Body Roundness Index (BRI), a relatively new anthropometric measure that reflects fat distribution, has shown promise in predicting metabolic and cardiovascular conditions (6). Unlike the Body Mass Index (BMI), which only reflects weight relative to height, and waist circumference (WC), which lacks adjustment for stature, BRI integrates height and waist circumference to capture body roundness and central adiposity in a standardized manner. This feature allows BRI to provide additional value beyond BMI and WC in evaluating cardiometabolic and cerebrovascular risk. However, despite growing interest in these individual markers, few studies have prospectively examined whether a vascular imaging marker and an anthropometric index jointly improve prediction through an interaction (product) term, particularly among elderly Chinese adults, leaving an important evidence gap.

From a clinical perspective, carotid stenosis is managed using a combination of best medical therapy, carotid endarterectomy, and carotid artery stenting, depending on stenosis severity, symptom status, and peri-procedural risk (7). In parallel, artificial intelligence has shown promise in the characterization of acute ischemic stroke subtypes and in supporting early decision-making (8), underscoring the broader movement toward multimodal and data-driven risk stratification in cerebrovascular disease. Recent systematic reviews and meta-analyses further suggest that BRI is associated with cardio-metabolic and cerebrovascular outcomes, often providing incremental value beyond BMI and WC, supporting its inclusion when exploring enhanced risk models (9, 10). Biologically, obesity-related systemic inflammation, insulin resistance, adverse lipid profiles, and endothelial dysfunction may accelerate atherosclerosis and plaque vulnerability, providing a plausible basis for a synergistic effect between central adiposity (captured by BRI) and carotid plaque burden on stroke risk (11).

In this prospective cohort study involving older adults, we examined the predictive value of maximum carotid plaque thickness and BRI in relation to the risk of stroke or TIA. To address the above knowledge gap, we explicitly modeled an interaction as the product of BRI and maximum carotid plaque thickness and evaluated whether this combined term enhances one-year stroke/TIA risk prediction beyond each marker alone. We hypothesized that the product term would yield superior discrimination compared with either component individually.

## 2 Methods

### 2.1 Study design

This was a prospective cohort study designed to assess the predictive value of carotid plaque thickness, BRI, and their interaction for the occurrence of stroke or TIA. Participants were consecutively recruited from elderly adults undergoing routine health examinations at the Physical Examination Center of Binzhou People’s Hospital between July 2022 and January 2024. The clinical study was approved by the ethical review board of Binzhou People’s Hospital. All procedures were conducted in accordance with the ethical standards of the Declaration of Helsinki. Written informed consent was obtained from all participants or their legally authorized representatives.

### 2.2 Inclusion and exclusion criteria

#### 2.2.1 Inclusion criteria

Age≥60 years; completed carotid ultrasound with measurements of maximum plaque thickness (mm) and maximum plaque length (mm); availability of height, weight, and waist circumference measurements allowing for BRI calculation; at least 1 year of follow-up with complete documentation of stroke or TIA occurrence.

#### 2.2.2 Exclusion criteria

Previously diagnosed with severe cardiovascular diseases (e.g., acute myocardial infarction, NYHA class III–IV heart failure); diagnosed with malignancies or severe malnutrition that could affect body composition measurements; severe carotid stenosis (≥70%) or prior carotid artery stenting; lost to follow-up due to illness or other reasons during the study period.

### 2.3 Variable measurement and definitions

#### 2.3.1 Carotid plaque assessment

All participants underwent high-resolution B-mode ultrasonography (Philips EPIQ7C, USA) conducted by the same experienced sonographer. The following measurements were obtained: ① Maximum plaque thickness: the greatest vertical thickness of any plaque detected in the common carotid artery, internal carotid artery, or at the carotid bifurcation. Based on prior studies, maximum plaque thickness has been widely validated as a reliable predictor of stroke risk and was therefore chosen as the primary imaging marker in our analysis. ② Maximum plaque length: the longest axial dimension of a single plaque. However, the clinical utility of plaque length has been reported as limited in previous literature, and thus it was not used as a primary endpoint in our study. All ultrasound measurements were performed by a single senior sonographer with more than 10 years of experience. Intra-observer reproducibility was evaluated in a subset of 30 participants, yielding an intra-class correlation coefficient (ICC) of 0.92 for maximum plaque thickness.

#### 2.3.2 BRI

BRI was proposed by Thomas et al. in 2013 as a novel anthropometric indicator to assess individual body fat distribution (12). The calculation formula is as follows: $\mathrm{BRI}=364.2-365.5\times \sqrt{1-{\left(\frac{\mathrm{WC}}{2\pi }\right)}^{2}÷{\left(0.5\times H\right)}^{2}}$ where WC represents waist circumference (in meters) and H denotes height (in meters). In this study, both waist circumference and height were measured using standardized procedures, then converted to meters before being applied in the formula.

#### 2.3.3 Interaction term

To investigate the combined effect of BRI and maximum plaque thickness, an interaction term (BRI × maximum plaque thickness) was computed. The interaction term was modeled as the direct product of the raw variables, rather than standardized values. This term was included in both logistic regression and ROC curve models to assess the added predictive value for stroke/TIA risk.

### 2.4 Endpoint definition

The primary endpoint was the occurrence of stroke or TIA within 1 year of follow-up, defined as follows: Stroke includes ischemic stroke (new infarct confirmed by cranial MRI) or hemorrhagic stroke (new intracerebral hemorrhage confirmed by cranial CT). TIA A transient episode of neurological dysfunction lasting less than 24 h without evidence of acute infarction on imaging. Exact event-time data were not collected; therefore, outcomes were analyzed as binary endpoints (occurrence vs. non-occurrence within 1 year) rather than as time-to-event data.

### 2.5 Statistical analysis

Statistical analysis was performed using R software (version 4.2.0), with significance set at *p* < 0.05. Continuous variables were compared using independent t-tests or Mann–Whitney U tests, while categorical variables were assessed using chi-square or Fisher’s exact tests. Pearson correlation coefficients with 95% confidence intervals were calculated to evaluate associations with stroke/TIA. Multivariable logistic regression was used to examine the independent effects of maximum plaque thickness, BRI, and their interaction term. Covariates were selected *a priori* based on prior literature and clinical relevance as potential confounders (including age, sex, prior stroke, hypertension, diabetes, dyslipidemia, and smoking). Two models were constructed: Age- and sex-adjusted model and Full multivariable adjustment model (further adjusted for prior stroke, hypertension, diabetes, dyslipidemia, and smoking). All key variables were fully recorded at baseline, and no missing data occurred; therefore, no imputation or case exclusion was required. ROC curve analysis was conducted to assess predictive performance, including AUC, optimal cutoff, sensitivity, and specificity. AUCs were compared using DeLong’s test to evaluate the added value of the interaction term. To assess potential model optimism, internal validation was performed using bootstrap resampling (1,000 iterations), and optimism-corrected AUC, calibration slope, and Brier score were reported.

## 3 Results

### 3.1 Baseline characteristics

A total of 230 elderly participants were included in this study, among whom 19 cases (8.26%) experienced stroke or TIA (Supplementary Figure S1). No significant age difference was observed between the groups (*p* = 0.968). The proportion of males was significantly higher in the stroke group compared to the non-stroke group (78.947% vs. 49.763%, *p* = 0.015). A higher prevalence of prior stroke was also observed in the stroke group (21.053% vs. 6.635%, *p* = 0.025). Hypertension was more common in the stroke group compared to the non-stroke group (84.211% vs. 40.758%, *p* = 0.032). No significant differences were found in smoking history, alcohol consumption, diabetes, coronary heart disease, or hyperlipidemia (all *p* > 0.05). The stroke group had significantly greater maximum plaque thickness (3.305 ± 0.515 mm vs. 2.245 ± 0.820 mm, *p* < 0.001) and BRI (4.872 ± 1.240 vs. 3.751 ± 0.916, *p* < 0.001). No significant difference was noted in maximum plaque length (*p* = 0.897) (Table 1).

**Table 1:** Baseline characteristics of study participants.

Variable	Non-stroke group (*n* = 211)	Stroke group (*n* = 19)	*p*-value
Age (years)	75.100 ± 9.228	74.474 ± 7.648	0.968
Sex (%)			0.015
Female	106 (50.237%)	4 (21.053%)	
Male	105 (49.763%)	15 (78.947%)	
Smoking history (%)			0.126
No	147 (69.668%)	10 (52.632%)	
Yes	64 (30.332%)	9 (47.368%)	
Alcohol consumption (%)			0.303
No	166 (78.673%)	13 (68.421%)	
Yes	45 (21.327%)	6 (31.579%)	
Hypertension (%)			0.032
No	86 (40.758%)	3 (15.789%)	
Yes	125 (59.242%)	16 (84.211%)	
Diabetes (%)			0.110
No	104 (49.289%)	13 (68.421%)	
Yes	107 (50.711%)	6 (31.579%)	
Coronary heart disease (%)			0.918
No	201 (95.261%)	18 (94.737%)	
Yes	10 (4.739%)	1 (5.263%)	
Hyperlipidemia (%)			0.567
No	187 (88.626%)	16 (84.211%)	
Yes	24 (11.374%)	3 (15.789%)	
Prior stroke (%)			0.025
No	197 (93.365%)	15 (78.947%)	
Yes	14 (6.635%)	4 (21.053%)	
Maximum plaque thickness (mm)	2.245 ± 0.820	3.305 ± 0.515	<0.001
Maximum plaque length (mm)	9.379 ± 5.547	9.942 ± 7.039	0.897
BRI	3.751 ± 0.916	4.872 ± 1.240	<0.001

### 3.2 Correlation analysis for stroke/TIA

As shown in Table 2, Pearson correlation analysis revealed that maximum plaque thickness (*p* < 0.001) and BRI (*p* < 0.001) were positively correlated with stroke/TIA. In addition, sex (*p* = 0.0146) and prior stroke history (*p* = 0.0250) also demonstrated significant positive associations. Interestingly, hypertension was negatively correlated with stroke/TIA (*p* = 0.0324). No significant correlations were observed for age, smoking, alcohol consumption, diabetes, coronary heart disease, hyperlipidemia, or maximum plaque length (all *p* > 0.05).

**Table 2:** Correlation analysis between stroke/TIA and clinical variables.

Variable	Correlation coefficient (95% CI)	*p*-value
Sex	0.1608 (0.0321, 0.2843)	0.0146
Age	−0.019 (−0.148, 0.1106)	0.7746
Smoking history	0.1008 (−0.029, 0.2272)	0.1276
Alcohol consumption	0.0679 (−0.062, 0.1956)	0.3049
Hypertension	−0.1411 (−0.2656, −0.012)	0.0324
Diabetes	−0.1054 (−0.2316, 0.0243)	0.1111
Coronary heart disease	0.0068 (−0.1227, 0.136)	0.9188
Hyperlipidemia	0.0378 (−0.092, 0.1663)	0.5689
Prior stroke	0.1478 (0.0188, 0.2719)	0.0250
Maximum plaque thickness (mm)	0.344 (0.2246, 0.4532)	<0.001
Maximum plaque length (mm)	0.0274 (−0.1023, 0.1562)	0.6791
BRI	0.3115 (0.1898, 0.4238)	<0.001

### 3.3 Logistic regression analysis

Multivariate logistic regression revealed that both maximum plaque thickness and BRI were independently associated with an increased risk of stroke/TIA across all models. Detailed regression coefficients and confidence intervals are provided in Table 3. In the Age- and sex-adjusted model, a 1 mm increase in maximum plaque thickness was associated with a 167.4% elevation in stroke/TIA risk (OR = 2.674, 95%CI:1.582–4.521, *p* = 0.00024), and the effect remained robust in the Full multivariable adjustment model, with an OR of 3.619 (95%CI: 1.781–7.355, *p* = 0.00038). BRI also maintained statistical significance in both models, showing a comparable increase in risk (Adjust I: OR = 2.742; Adjust II: OR = 3.116; all *p* < 0.001). In contrast, maximum plaque length did not demonstrate a significant association in either model (*p* > 0.05).

**Table 3:** Logistic regression analysis of carotid plaque characteristics and BRI in predicting stroke/TIA.

Variable	Age- and sex-adjusted model OR (95% CI), *p*-value	Full multivariable adjustment model OR (95% CI), *p*-value
Max. plaque thickness (mm)	2.674 (1.582, 4.521), 0.00024	3.619 (1.781, 7.355), 0.00038
Max. plaque length (mm)	0.992 (0.909, 1.083), 0.86310	0.975 (0.878, 1.083), 0.63834
BRI	2.742 (1.722, 4.366), 0.00002	3.116 (1.784, 5.444), 0.00006

Age- and sex-adjusted model = adjusted for age and sex; Full multivariable adjustment model = further adjusted for prior stroke, hypertension, diabetes, dyslipidemia, and smoking, based on established confounders reported in prior studies.

### 3.4 ROC curve analysis

**Figure 1:**
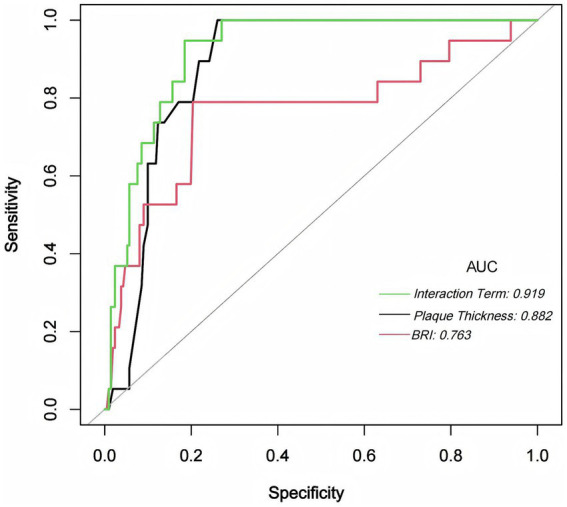
ROC Curves for BRI, Maximum Plaque Thickness, and Their Interaction Term. This figure illustrates the ROC curves for BRI (red), maximum carotid plaque thickness (black), and their interaction term (green) in predicting stroke or TIA. The interaction model yielded the highest AUC (0.919), followed by maximum plaque thickness (AUC = 0.882) and BRI (AUC = 0.763). The combined model provided the strongest overall discrimination, particularly compared with BRI. Its advantage over maximum plaque thickness was numerical but did not reach statistical significance (see Supplementary Table S4).

ROC analysis was conducted to evaluate the predictive performance of maximum carotid plaque thickness, BRI, and their interaction term (BRI × maximum plaque thickness) for the occurrence of stroke or TIA. The interaction model demonstrated the highest predictive accuracy with an AUC of 0.9192 (95% CI: 0.8772–0.9612), an optimal cutoff value of 10.8232, a specificity of 0.8152 (95% CI: 0.74–0.87), and a sensitivity of 0.9474 (95% CI: 0.74–0.99). The AUC for maximum carotid plaque thickness alone was 0.8819 (95% CI: 0.8353–0.9285), with an optimal cutoff value of 2.4500, specificity of 0.7393 (95% CI: 0.66–0.81), and sensitivity of 1.0000 (95% CI: 0.82–1.00). The AUC for BRI alone was 0.7632 (95% CI: 0.6266–0.8997), with an optimal cutoff value of 4.3282, specificity of 0.7962 (95% CI: 0.71–0.87), and sensitivity of 0.7895 (95% CI: 0.54–0.93). Pairwise AUC comparisons showed that the interaction model significantly outperformed BRI (bootstrap *p* < 0.001), whereas the difference versus maximum plaque thickness was not statistically significant, though the interaction model consistently demonstrated numerically higher discrimination (Figure 1). After bootstrap correction (B = 1,000), the optimism-corrected AUC of the interaction model was 0.897 (95% CI: 0.788–0.954), with a calibration slope of 0.972 and a Brier score of 0.059 (Supplementary Table S3). Detailed pairwise AUC comparison and CI for sensitivity and specificity are summarized in Supplementary Table S4.

## 4 Discussion

In this study, we evaluated how maximum carotid plaque thickness, BRI, and the interaction between the two relate to the likelihood of stroke or TIA. The data indicated that both plaque thickness and BRI were independently associated with elevated stroke risk, even after adjusting for a set of confounders selected on the basis of prior evidence and clinical relevance. More notably, the interaction between them demonstrated the highest discriminative value in ROC analysis (AUC = 0.9192, 95% CI: 0.8772–0.9612). Compared with BRI alone (AUC = 0.7632), the interaction model showed significantly better discrimination, while its advantage over maximum plaque thickness (AUC = 0.8819) was numerical but did not reach statistical significance. After bootstrap internal validation, the optimism-corrected AUC of the interaction model remained high at 0.897 (95% CI: 0.788–0.954), confirming the robustness of this predictive advantage. These findings point toward a possible compounding effect—where fat distribution and vascular pathology may act in concert—thereby underscoring the utility of a combined assessment model in clinical screening.

That carotid atherosclerosis is a major contributor to stroke is well-established. Specifically, maximum plaque thickness has long been considered a meaningful indicator for vascular risk (6, 13). Here, we found that stroke patients had markedly thicker plaques than non-stroke participants. Logistic regression models showed that each 1 mm increment in plaque thickness was associated with a 167.4% increase in stroke/TIA odds (OR = 2.674, 95%CI: 1.582–4.521, *p* = 0.00024), rising further after more extensive adjustment (OR = 3.619, 95%CI: 1.781–7.355, *p* = 0.00038). These observations resonate with prior reports—for example, Petrovic et al. (6) identified a threshold of 2.5 mm plaque thickness as clinically relevant, while Jumah et al. (14) linked plaques exceeding 3 mm to embolic strokes, even when luminal stenosis was absent. Abe et al. further demonstrated that subtle increases in carotid intima-media thickness (e.g., by 0.17 mm) were linked to a 30% rise in stroke hazard (15). While our findings affirm the importance of plaque thickness, they also show that plaque length, in contrast, bore no clear association (*p* = 0.6791). This may reflect its limited functional implication, as plaque composition—not just size—plays a central role in vulnerability (16, 17). Future studies incorporating advanced imaging modalities, such as high-resolution MRI or ultrasound elastography, will be important to capture plaque composition and vulnerability, which were not assessed in this study.

Interestingly, hypertension showed a negative correlation with stroke/TIA in univariate analysis. We believe this paradoxical result is most likely explained by baseline imbalances, residual confounding, or the small number of events, rather than indicating a true protective effect. This finding should therefore be interpreted with caution, as extensive evidence from large-scale studies consistently supports hypertension as a major risk factor for stroke.

The BRI, on the other hand, reflects body shape more precisely than BMI and may capture hidden metabolic risks (18, 19). Compared with BMI, which cannot distinguish fat distribution, and WC, which does not account for height differences, BRI provides a more comprehensive indicator of central adiposity. This methodological advantage may partly explain why BRI showed independent predictive value in our cohort and outperformed BMI and WC in prior studies of vascular and metabolic outcomes. In our cohort, BRI showed independent predictive value for stroke/TIA (Full multivariable adjustment OR = 3.116, *p* = 0.00006), with an AUC of 0.7632, which supports its potential as a screening tool. This aligns with research by Peng et al. (20), who reported that individuals with high BRI had a significant increase in odds of stroke (HR = 1.158, 95%CI:1.158–1.159). Similarly, Cai et al. (21) found BRI to outperform traditional obesity indices like BMI in forecasting vascular events, reinforcing its emerging relevance. From a biological perspective, obesity-related chronic inflammation may accelerate atherosclerosis by promoting systemic inflammatory responses (e.g., CRP, IL-6), increasing plaque vulnerability and thereby compounding stroke risk. This mechanism may explain the stronger predictive value of the interaction between BRI and plaque thickness observed in our study.

What sets this study apart is the demonstration that combining BRI and plaque thickness improves risk prediction (22, 23). The synergistic model yielded the highest AUC, implying that fat distribution might accelerate the development of atherosclerosis, thereby raising stroke risk. This is not entirely new, as other researchers have made similar observations: for example, Fu et al. (24) noted a stronger plaque–stroke link in obese populations (HR = 4.1), and Ozbeyaz et al. (25) reported elevated inflammation and increased carotid wall thickness in obese individuals. Inflammatory processes, as highlighted by Gong et al. (26), also appear to play a key role; their study linked residual inflammatory risk to vulnerable plaque features. Taken together, these findings suggest that assessing carotid pathology in isolation might overlook important metabolic contributors. Including metrics like BRI in risk evaluation—particularly in patients with metabolic syndrome—could allow for earlier and more personalized interventions.

Nonetheless, this study is not without limitations. First, the relatively small sample size (*n* = 230) and limited number of outcome events (*n* = 19) resulted in a low events-per-variable ratio, which may reduce statistical power and increase the risk of overfitting. We addressed this by conducting bootstrap internal validation, but the findings should still be interpreted cautiously and validated in larger, multicenter cohorts. Second, because the exact timing of outcome events was not collected, analyses were restricted to binary endpoints (occurrence vs. non-occurrence within 1 year), rather than time-to-event models. This may limit the ability to fully capture the dynamics of stroke risk over time. Third, prior stroke was significantly more frequent in the stroke group. Although this variable was adjusted for in our full multivariable model, we were unable to stratify primary and recurrent strokes separately due to the small number of events. This limitation should be addressed in larger studies. Fourth, the follow-up duration of only 1 year is relatively short for stroke risk prediction, and longer-term follow-up would provide a more comprehensive understanding of risk trajectories. Fifth, we did not evaluate the internal composition of plaques, which could influence their vulnerability and stability. Future work incorporating advanced imaging modalities could address this gap.

Moreover, the cutoff values identified from ROC analyses (e.g., plaque thickness of 2.45 mm, BRI of 4.33, and interaction value of 10.82) may provide preliminary guidance for early risk stratification. However, we emphasized in the revised text that these thresholds should be interpreted as exploratory, given the small event number, and require validation in independent cohorts before being applied clinically.

In sum, both maximum plaque thickness and BRI offer independent predictive value for stroke, and combining them provides even stronger discrimination. Importantly, our findings suggest potential for incorporating these measures into conventional risk scores (e.g., Framingham or ASCVD) to enhance early risk stratification. This could guide targeted screening and preventive interventions, particularly in individuals with metabolic syndrome or subclinical carotid atherosclerosis.

## Data Availability

The raw data supporting the conclusions of this article will be made available by the authors, without undue reservation.
