# Supplementary figures and images for: Predictive value of the product term BRI × carotid plaque thickness for stroke and transient ischemic attack: a prospective cohort study
Source: Front Neurol. 2025 Sep 17;16:1622941. doi: 10.3389/fneur.2025.1622941 (PMC12483859; doi:10.3389/fneur.2025.1622941)

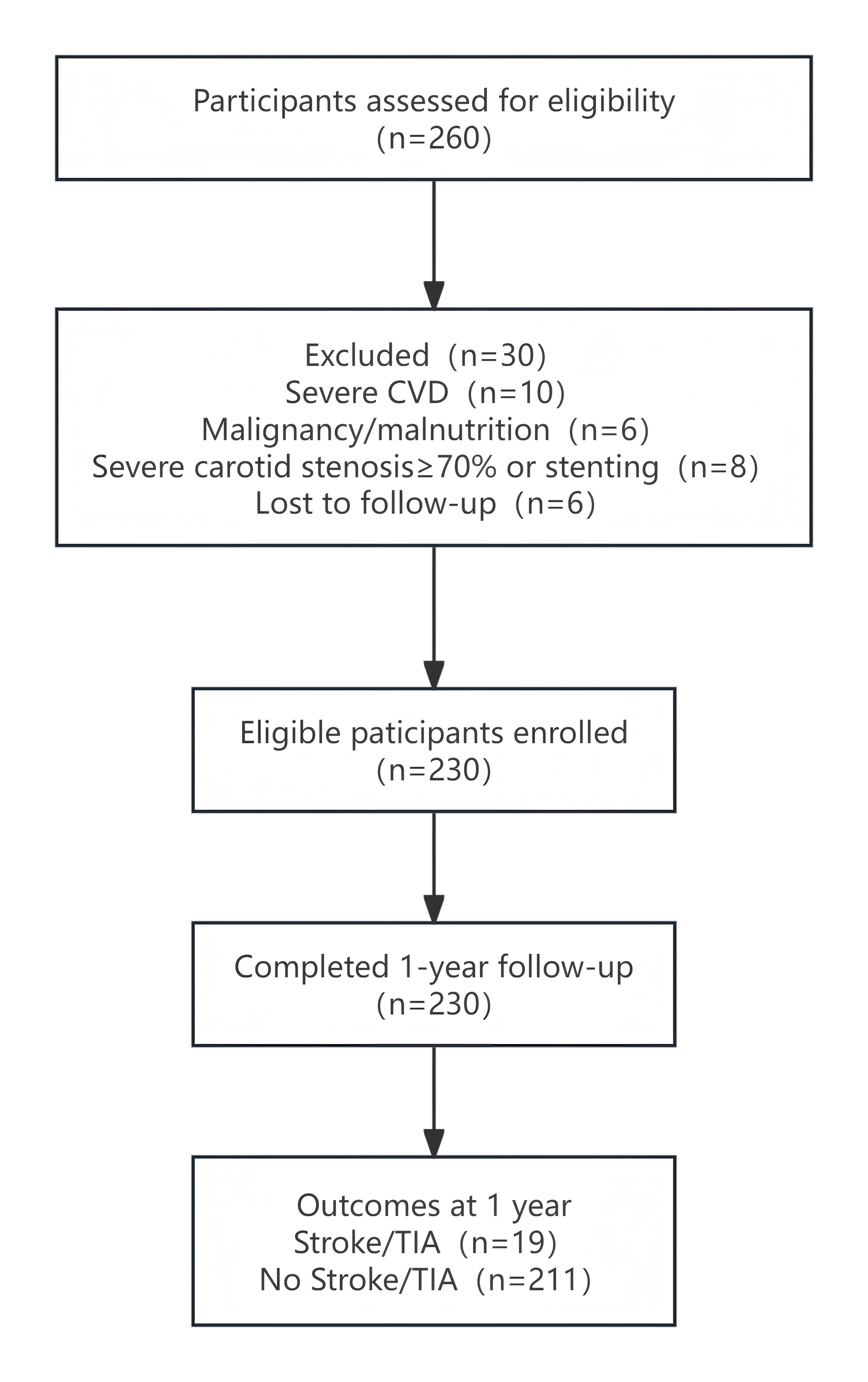

Supplement: Supplementary file 1 [file Image_1.jpg]
