# Supplementary material for: Predictive value of the product term BRI × carotid plaque thickness for stroke and transient ischemic attack: a prospective cohort study
Source: Front Neurol. 2025 Sep 17;16:1622941. doi: 10.3389/fneur.2025.1622941 (PMC12483859; doi:10.3389/fneur.2025.1622941)
Supplement: Supplementary file 3 [file Table_2.DOCX]

**Supplementary Table S2. Pairwise comparisons of AUCs between models**

| **Comparison** | **AUC difference (95% CI)** | **p-value** |
| --- | --- | --- |
| Interaction vs. Maximum plaque thickness | 0.037 (−0.013 to 0.085) | 0.144 |
| Interaction vs. BRI | 0.156 (0.041 to 0.293) | <0.001 |

**Legend:** Pairwise AUC comparisons were performed using bootstrap resampling (B=1000). The interaction model significantly outperformed BRI, while its advantage over maximum plaque thickness was numerical but did not reach statistical significance.
