# Supplementary material for: Predictive value of the product term BRI × carotid plaque thickness for stroke and transient ischemic attack: a prospective cohort study
Source: Front Neurol. 2025 Sep 17;16:1622941. doi: 10.3389/fneur.2025.1622941 (PMC12483859; doi:10.3389/fneur.2025.1622941)
Supplement: Supplementary file 4 [file Table_3.docx]

**Supplementary Table S3. Internal validation results of predictive models**

| **Model** | **Apparent AUC** | **Optimism-corrected**  **AUC (95% CI)** | **Calibration slope** | **Brier score** |
| --- | --- | --- | --- | --- |
| Maximum plaque  thickness | 0.882 | 0.872 (0.754–0.936) | 0.968 | 0.063 |
| BRI | 0.763 | 0.752 (0.613–0.869) | 0.954 | 0.071 |
| Interaction term (BRI×plaque thickness) | 0.919 | 0.897 (0.788–0.954) | 0.972 | 0.059 |

**Legend:** Internal validation was conducted using bootstrap resampling (B=1000). Apparent AUC, optimism-corrected AUC with 95% CI, calibration slope, and Brier score are reported for each model to assess predictive performance and robustness.
